# K-Mer Spectrum-Based Error Correction Algorithm for Next-Generation Sequencing Data

**DOI:** 10.1155/2022/8077664

**Published:** 2022-07-14

**Authors:** Hussah N. AlEisa, Safwat Hamad, Ahmed Elhadad

**Affiliations:** ^1^Department of Computer Sciences, College of Computer and Information Sciences, Princess Nourah bint Abdulrahman University, Riyadh, Saudi Arabia; ^2^Department of Scientific Computing, Faculty of Computer and Information Sciences, Ain Shams University, Cairo, Egypt; ^3^Department of Computer Science, Faculty of Computers and Information, South Valley University, Qena, Egypt

## Abstract

In the mid-1970s, the first-generation sequencing technique (Sanger) was created. It used Advanced BioSystems sequencing devices and Beckman's GeXP genetic testing technology. The second-generation sequencing (2GS) technique arrived just several years after the first human genome was published in 2003. 2GS devices are very quicker than Sanger sequencing equipment, with considerably cheaper manufacturing costs and far higher throughput in the form of short reads. The third-generation sequencing (3GS) method, initially introduced in 2005, offers further reduced manufacturing costs and higher throughput. Even though sequencing technique has result generations, it is error-prone due to a large number of reads. The study of this massive amount of data will aid in the decoding of life secrets, the detection of infections, the development of improved crops, and the improvement of life quality, among other things. This is a challenging task, which is complicated not just by a large number of reads and by the occurrence of sequencing mistakes. As a result, error correction is a crucial duty in data processing; it entails identifying and correcting read errors. Various k-spectrum-based error correction algorithms' performance can be influenced by a variety of characteristics like coverage depth, read length, and genome size, as demonstrated in this work. As a result, time and effort must be put into selecting acceptable approaches for error correction of certain NGS data.

## 1. Introduction

Nature methods named next-generation high-throughput DNA sequencing techniques as the method of the year in 2007. These methods are creating interesting new potential in biology [1]. The road to garnering the approval of the revolutionary technology, on the other hand, was not simple. Until recently, the Sanger enzymatic dideoxy method, first explained in 1977, and the Maxam and Gilbert chemical degradation technique, first mentioned in the same year, were the methodologies used for sequence analysis. The Maxam and Gilbert chemical degradation technique was used in sequential cases that could not be solved easily with the Sanger method [2]. The potential to decipher genomes and conduct ground-breaking biomedical sciences has been made possible by the rapid synthesis and accessibility of enormous amounts of DNA sequencing obtained by next-generation sequencing (NGS) technology at a lower cost than traditional Sanger sequencing [3]. There has been a significant trend apart from using automated Sanger sequencing for genome analysis in the last four years. Previous to this departure, the automated Sanger sequencing had taken over the market for half a century, resulting in a slew of significant achievements, such as the production of the only completed human genome sequence.

Despite numerous technological advances during this period, the drawbacks of automated Sanger sequencing demonstrated the need for new and superior methods for sequencing huge numbers of human genomes [4]. Sanger sequencing has seen less documented advancements as recent attempts have been focused on the development of novel technologies [5]. As a result, automated Sanger sequencing is not addressed in this paper, and curious readers should go to earlier pages. The mechanized Sanger approach is a “first-generation” approach, whereas “next-generation sequencing” (NGS) refers to newly developed technology. A range of methods combining templates' sequencing, processing, but also imaging, and also genomic alignments and assembling approaches are included in these modern technologies [6]. The introduction of next-generation sequencing (NGS) techniques to the markets has altered how we thought regarding scientific methodologies in fundamental, practical, and healthcare findings. In certain ways, the promise of NGS is similar to that of PCR in the initial periods, with the major limitations being one's imagination [7]. The capability to create a tremendous volume of information for a low cost—in certain cases exceeding of one billion shorter readings each instrument's cycle—is NGS's biggest advantage. This component broadens the scope of exploration beyond defining basic ordering [8]. Microarrays, for instance, are being phased out of gene expressing research in favour of sequence-based approaches, which may detect and estimate uncommon transcripts without previous understanding of a gene and offer data on alternate sequencing and splicing variations in discovered genes [9].

The capacity to sequence the entire genomes of several closely connected organisms has enabled large-scale comparing and developmental investigations that were previously unthinkable [10]. The sequence analysis of individual genomes to improve our knowledge of how genetic variants impact diseases and health might be the most broad application of NGS [11]. Several platforms are expected to coexist in the industry due to the variety of NGS characteristics, with several providing apparent benefits for specific implementations over another [12]. Template development, sequential and visualization, and information processing are only a few of the processes used in sequenced techniques [13–16]. The kinds of information generated by every platform are determined by the distinctive combined effect of specialized standards that differentiates one technique from others [17]. When evaluating technologies depending on information cost and quality, these disparities in information production provide a hurdle. Even though every manufacturer provides performance ratings and precision predictions, there is no agreement that a ‘reliability foundation' from one platform was similar to that from others [18].

## 2. Related Works

Six k-spectrum-based approaches, namely Trowel, Reptile, Bloocoo, Musket, Lighter, and Bless, were evaluated utilizing six generated collections of paired-end Illumina sequencing information in this technique [19]. The genome size, coverage depth, and read length of these NGS databases changed. The Error Rectification Evaluation Toolkit (ECET) was used to provide a set of metrics (such as true positives, false positives, false negatives, recall, accuracy, gain, and F-score) for evaluating every programme's correcting performance. Musket exhibited the best total effectiveness throughout the spectrum of studied variations indicated in the six databases, according to findings from computational simulations. Musket's efficiency was lesser in a population with a moderate read length, coverage depth, and a short genome (F-score = 0.81). The remaining five techniques executed poorly (F-score <0.80) or refused to analyse one or many data sources. Individual k-spectrum-oriented error correction algorithms' effectiveness can be influenced by a variety of characteristics including such genome size, coverage depth, and read lengths, as demonstrated in this work. As a result, time and attention must be put into selecting acceptable approaches for error correction of particular NGS databases. Due to its continuously improved effectiveness through every six experimental databases, we propose Musket as the best choice depending on our comparison analysis.

Longer read error correction has become a concern for bioinformaticians, motivating the implementation of novel error correction algorithms tailored to NGS technology. We introduced a new approach for error correction of LLRs created by an NGS sequencing utilizing another LLRs produced by the similar NGS sequencing in this publication [20]. As a result, our method is self-correcting. They describe a novel de novo self-error correction technique that uses just lengthy reads in this study. Our technique is divided into two parts: to begin, they employ a quick hashing algorithm that enables us to locate connections among the largest reads as well as other reads in a collection of extensive reads. Then, utilizing a dynamic programming approach in a band of width *w*, they employ the largest reads as seeds to determine the overall alignments of long reads. In contrary to previous hybrid error correction algorithms, the error correction technique does not require high-quality reads. They are now doing an experimental investigation on our self-correction method. They would utilize fictional information in which they have developed and actual information produced by Oxford Nanopore and Pacific Biosciences sequencers to conduct this research. The findings would be compared with those achieved by other self-correction methods.

They provide two novel effective error correction methods tailored for viral amplicons in this study [21]: (a) k-mer-based error correction (KEC) and (b) empirical frequency threshold (ET). Both were tested against a recently reported clustering technique (SHORAH) on 25 testing databases generated by 454-sequencing of amplicons with specified sequencing. Discovering actual haplotypes is comparable for all three techniques. ET and KEC, on the other hand, were far more effective than SHORAH at eliminating erroneous haplotypes and determining the frequencies of actual haplotypes. Both the ET and KEC techniques are well suited to the quick extraction of error-free haplotypes achieved from 454-sequencing of amplicons from heterogeneous viruses. In terms of detecting actual KEC, SHORAH, haplotypes, and ET are all similarly effective. Meanwhile, newer methods, ET, and KEC are more effective than SHORAH at eliminating erroneous haplotypes and determining the frequencies of actual haplotypes. Both techniques are well suited to quickly recovering high-quality haplotypes from reads generated by NGS of amplicons from diverse viruses like HIV and HCV.

In this study [22], two distinct error-correcting types of software, ECHO and Quake, are tested on next-generation sequencing information from heterozygous genomes to assess how well they function. Quake and ECHO performed admirably and were capable to rectify most of the information's problems. Errors that occurred in heterozygous sites, on the other hand, have a distinct pattern. Inaccuracies at these locations were occasionally wrongly rectified, bringing errors into the database and perhaps resulting in a chimeric read. The quake had a substantially lower chance of producing chimeric readings. Read cutting in Quake deleted a significant amount of the original information, leaving reads with fewer heterozygous markers. ECHO produced more chimeric reads and generated more mistakes than Quake, but heterozygous markers were maintained. The assembling statistics were enhanced by utilizing genuine *E. coli* sequenced information and their assemblies following error correction. It was also discovered that sorting reads by haplotype improves assembling performance. These results imply that when employed to heterozygous information, both ECHO and Quake have benefits and drawbacks. With the growing popularity of haplotype-specific research, newer technologies that are meant to be haplotype-aware and do not have the flaws of ECHO and Quake are required.

They introduce Karect and unique multidimensional alignment-based error-correcting approach for next-generation sequencing information [23]. Replacement, addition, and removal errors are all supported by our method. Karect is dependent on multiple orientations; therefore, it can manage nonuniform coverage and also somewhat occupied sections. It also allows substitutions, inserting, and deleting mistakes. This could manage nonuniform coverage and also portions of the sequencing genomes that are only partly covered. Tests using data sets from 454 FLX, Ion Torrent, and Illumina sequenced machinery show that Karect is more efficient than earlier approaches in both fixing individual dependent errors (up to a 10 per cent improvement in efficiency gains) and post-de novo assembling efficiency (up to 10 per cent enhancing in NGA50). They also present a new paradigm for assessing the accuracy of error corrections. Karect provides improved error-correcting comparison to current state-of-the-art approaches, according to comprehensive experimental assessment. When employed as a preprocessing phase for newer assemblers, Karect also results in much enhanced components. They presently do not handle Pacific Biosciences data due to chimeric readings; however, they are functioning to resolve this.

To reduce the impact of mistakes on the detection of minority variants, they established a probabilistic Bayesian strategy [24]. Pyrosequencing information from a 1.5-kb segment of the HIV-1 gag/pol gene in two controls and two medical samples was used to test it. The impact of PCR amplification was looked into. In the PCR-amplified and non-PCR data sets, error correction reduced the pyro sequenced based substitution rates by two and five times, correspondingly, from 0.05 per cent to 0.03 per cent and from 0.25 per cent to 0.05 per cent. With complete sequencing reconstructions, they were capable to discover viral clones as rare as 0.1 per cent. In terms of recall and precision, probabilistic haplotype inference exceeds counting-based identification. The genetic diversity found inside and among two medical data sets resulting in a variety of phenotypic drug-resistant characteristics, implying a strong epidemiological relationship. They conclude this, if technological problems are appropriately addressed, pyrosequencing could be utilized to analyse genetically heterogeneous materials with great efficiency.

## 3. Methodology

**Figure 1:**
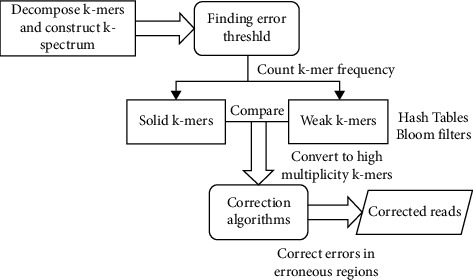
Framework for the proposed method.

Error correction approaches depending on k-spectrum are derived from prior spectral alignments implementations of de Bruijn graph assemblers and following an extended architecture as illustrated in Figure 1. In a collection of readings, a k-spectrum is the probability of a collection of decomposing separate substrings of length *k* (in other words, k-mer). Within the spectra characteristic area, it estimates the presence of every k-length contiguous strings expressed as vectors. When contrasted to sequencing without errors, errors in sequencing should result in a large diverging at low k-mer frequency. Inconstant genomic repetitions and genome sampling can arise at high frequencies in mistake correction, resulting in a plethora of identically susceptible correction alternatives. This means that k-mers with short hamming distances are likely from the identical genomic region and must be adjusted depending on their frequency of occurrences. After extracting k-mers from sequenced reads, the k-spectrum-based correction begins by providing a weighed number to every k-mer. Depending on categorized counting frequency or basic performance ratings, the number is provided. Weak (untrusted or insolid) k-mers with lower frequency are differentiated from solid (trusted) k-mers by evaluating and setting an appropriate error threshold (with higher frequency). Error correction is applied to reading with weak k-mers by continuously transforming those to solid k-mers until there are no more weakly k-mers in the sequencing. After validation, only solid k-mers would be maintained.

### 3.1. Bloom Filter

Bloom filtering is used as the data framework in the bulk of the approaches explored in this research. A Bloom filtering is a space-effective probability information framework that uses binary arrays and several hash operations to determine whether an entry is a component of a subset. This could correctly identify a collection's nonmember component. A Bloom filtering has a 100 percentage recall probability because a query might generate false positives, however no false negatives. A Bloom filtering does not save the components themselves, but it does enable you to see whether an object is definitely missing from the Bloom filtering or if it has been included to the Bloom filtering. Most approaches use the counted Bloom filter variation for sequencing error corrections, wherein array locations are not individual values but an n-bit counter. The amount of bits in the arrays, the number of hashing algorithms, and, more significantly, the reliability of the hash processes all affect the effectiveness of Bloom filtering.

### 3.2. KEC Algorithm

The k-mer-based error correction (KEC) system consists of four phases:

1. Determine the number of k-mers *s* and their frequency *kc*(*s*) (k-counts). We presume that k-mers with a high k-count (“solid”) are right, but k-mers with a low k-count (“weak”) include errors.
2. Establish the k-count threshold (threshold error) that separates solid k-mers from weak k-mers.
3. Locate the error locations. The read's error zone is the section [*i*,  *j*], where the k-mer beginning at position *p* is evaluated weak for each [*i*,  *j*].
4. Errors in error sections should be corrected. Let *r* = (*r*_1_, ...,  *r*_*n*_) be the constant reads, and *r*_*i*_ ∈ {*A*,  *T*,  *G*,  *C*} be the variable read. *S*_*k*_(*i*) denotes the k-mer of *r* beginning at location *i*, and *KC*_*k*_(*i*) is the k-count of this k-mer. Let pref_*j*_(*s*) be the prefixed of length *j* of an unspecified sequence *s*.

#### 3.2.1. Calculating K-Mers and K-Counts

The individual reads *r*, as well as their frequency *f*_*r*_, were saved. Due to the often huge size of the knowledge collection, simple calculations of k-mers and k-counts are ineffective. We utilize a hashed map with every key being a k-mer *s* and the value being the arrays *v*(*s*) = (*r*,  *i*) :  *s* = *S*_*k*_(*i*) in the read *r*. Including very huge database collections, the hash mapping could be quickly created.

#### 3.2.2. Finding the Error Threshold

To determine the error threshold, the concept suggested is employed considering the frequency distributions of k-count variables. The frequency of the k-count integer *v* is denoted by *f*(*v*). The k-counts of erroneous k-mers and accurate k-mers are considered to have distinct probabilities. It was discovered that the prototype for the distributions does not need to be directly considered because the initial minimum of *f*(*v*) satisfactorily differentiates distinct probabilities and could thus be utilized as the error threshold. Furthermore, due to the more discontinuous distributions of k-count readings in amplicon information than in shotgun trials, this technique is frequently inapplicable. The break in the distributed relates to the first minimum of *f*(*v*), which is normally equivalent to 0 (in other words, to the first k-count value, there is no equivalent k-mers). The error threshold *t*_*er*_ is defined as the end of the first suitably lengthy section of consecutive 0′*s* in *f*(*v*). The method's component is the duration of the section.

#### 3.2.3. Finding Error Regions

Each read's error regions are estimated as followed. We start by looking for independent sections [*i*,  *j*] in order to identify *KC*_*k*_(*p*) ≤ *t*_*er*_ for each and every *p* ∈ [*i*,  *j*]. The read's k-mers are then categorized as per their k-counts utilizing the varied bandwidth mean-shift technique of cluster analysis. We utilize the variable bandwidth mean-shift technique's fast deployment FAMS. Furthermore, each segment is expanded in both orientations by inserting sequential places *q* according to the accompanying rule: *q* is inserted if and only if *p* *ϵ* [*i*,  *j*] occurs and k-mers *S*_*k*_(*p*) and *S*_*k*_(*q*) correspond to the similar clustering. Overlapping sections are linked together, and the resultant sections are called error areas.

#### 3.2.4. Error Correction

There are three components in this phase:

- Correction of errors in “short” error areas (with lengths not exceeding *k*)
- Correction of errors in “long” error areas (with lengths higher than *k*)
- Postprocessing and reconstruction of haplotypes

Phases (a) and (b) can be applied to every sequenced data and are regarded independent algorithms. The amplicon information is processed in phase (c).

Instruments for assessment to take advantage of its neutral structure recognized as targeted error formats (TEF), and the Error Correction Evaluation Toolkit (ECET) versions were employed for effectiveness assessment. ECET also generates error correction data and measurements, which could be utilized to evaluate efficiency immediately. The discrepancy between the erroneous sequence and the reference genome was determined by read alignments. The conversion of SAM (sequence alignment/mapping) structured data to FASTQ documents performed by the sam-analysis.py script in ECET was validated using SamToFastq, one of the Picard command line instruments.

**Figure 2:**
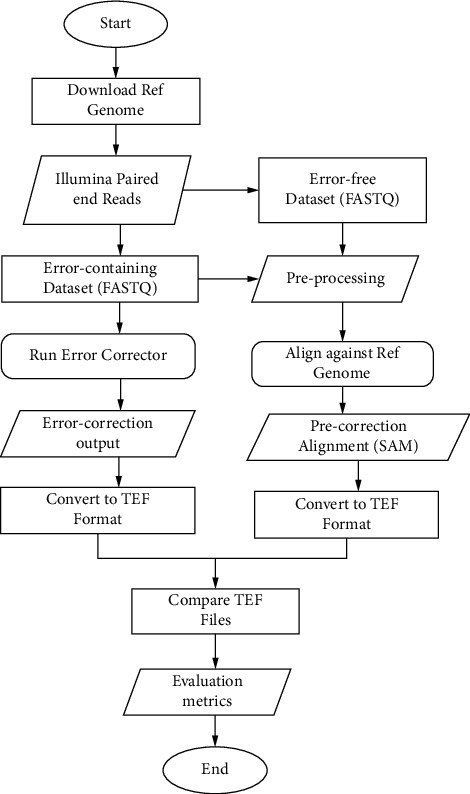
Flowchart for the proposed method.

Sequencing database simulations, postcorrection, and precorrection alignment to reference genomes and derivation of assessment measures and statistics are all processes in the approach, as illustrated in Figure 2. In a nutshell, FASTQ format was used to construct both error-containing paired-end and error-free sequencing. During the procedure, the error-free information was used for QA/QC. Simulated sequences were associated with a reference genome utilizing BWA afterwards translating FASTQ to FASTA (preprocess owing to ECET's header demands before alignment). ECET was used to transfer the SAM alignments documents generated by BWA to TEF format. Error-infested statistics were cleaned up with error-correcting software. ECET was used to transform the error correction findings from these instruments to TEF documents. The Comp2PCAlign tool included in ECET was used to evaluate the TEF records that produced postcorrection and precorrection measurements and statistics for effectiveness evaluation.

## 4. Experimental Results

The effectiveness measures that were obtained are shown in the tables. A negative gain number indicates that additional errors are generated than rectified in the data set. Negative gains were seen in five approaches (Bless, Trowel, Bloocoo, Lighter, and Reptile), particularly in the synthesis of EC-3. The most thorough measurement of mistake correcting effectiveness is the F-score. With the exception of Musket, every approaches outperformed with at least one data set when F-score = 0.96 was used as the threshold for satisfactory efficiency. As a result, Musket had the total finest efficiency, while Trowel had the lowest, with five examples of poor effectiveness.

### 4.1. Analysis of the Context of the Error Correction Base Sequence

We looked at the sequenced tuples surrounding error locations to see whether there were any preferences for sequencing composing near them. We selected the appropriate section of the reference sequencing for the study because errors at location 1 do not have previous bases and errors at the place last do not have subsequent characters in the reads. This also prevents the analysis of incorrect base calls in error-prone read sequencing near to the mistake site in question. Since this section of the source sequences is not section of the sequencing fragments, the sequencing component preceding the read beginning is not recognized to be the source of an error at position 1. The bases succeeding the conclusion of the read, on the other hand, may have an impact on base calling. We chose to search for the surrounding bases in the reference sequencing for every mistake locations in the similar way. We did not regard every tuples in the referenced sequencing as referenced tuples, but rather every tuples across all individually paired reads (added 6 bases preceding and following the relevant read section from the reference sequencing). This is done to avoid read coverage bias towards GC-rich sections of the reference sequenced in the study. We produced sequencing logos for Beta and *Helicobacter* using comparative frequency for 4 to 12 base tuples encompassing the mistake in the centre location. We exhibit the 4 and 6 bases tuple findings in scatterplots with the tuple frequency for all data set collections to visualize the overall pattern. In every information collections, all 3-base tuples are beginning with a *G* are obviously dominant, with *G*-error-A and *G*-error-*G* becoming the leading choices. Errors containing tuples beginning with *A* *or* *T* are underestimated, but errors containing tuples beginning with *C* are as common as referenced tuples. *T* is the third base in the three least frequently tuples *T*-error-*T*, *C*-error-*T*, and *A*-error-*T*, and is the least recurring base following an error. In the scatterplot with 6-base tuples, the pattern of *G* becoming the most common base preceding an error is kept and emphasized even better. Before an error, *Gs* are usually the favoured bases, and *Ts* are the least commonly used bases. The incorrect location was preceded by *G* in 36 and 33 per cent of instances (*Helicobacter* and Beta, correspondingly).

### 4.2. Error Correction Techniques' Efficiency and Productivity

To test the efficiency and productivity of every error correction approach, we utilized a comprehensive collection of evaluating measures. True positives (TP) were described as errors that were properly resolved by the error correction instrument, false positives (FP) were characterized as appropriate bases that were wrongly modified by the instrument, false negatives (FN) were characterized as inaccurate bases that were not corrected or inaccurately resolved by the instrument, and true negatives (TN) were characterized as appropriate bases that were not impacted by the instrument. To evaluate the effectiveness of every error-correcting technology, we employed the gained measure [25]. A positive gain implies that the error correction procedure had a positive overall influence, whereas a negative gain suggests that the device performed more incorrectly than correctly. A gain of 1.0 indicates that the error correction tool performed completed required adjustments without affecting the FP. Precision was described as the percentage of appropriate corrections made out of the overall number of corrections made by the error correction instrument. Sensitivity measures the proportion of rectified errors between every detected error in the data set; in other terms, sensitivities show that techniques repair the greatest number of generated errors [25]. Lastly, we looked to see whether the error correction approaches removed bases from updated reads at the starting or finish. Reducing the bases could be part of an attempts to rectify a deletions (TP trimming), or it could just be a case of eliminating a proper base (FP trimming).

### 4.3. Correction of Errors and Reconfiguration of Haplotypes

The per-base error ratio for the non-PCR-amplified sample was 0.05 per cent, while the expanded collection had a greater error ratio of 0.30 per cent. As a result, ∼12 per cent of non-PCR reads have one or more sequenced errors, while 44 per cent of PCR reads have one or more sequenced errors. After the error correction technique, the error rate for the PCR and non-PCR instances lowers to 0.04 per cent and 0.06 per cent, correspondingly, resulting in ∼93 per cent error-free readings in both data sets.

### 4.4. Estimation of Frequency

**Figure 3:**
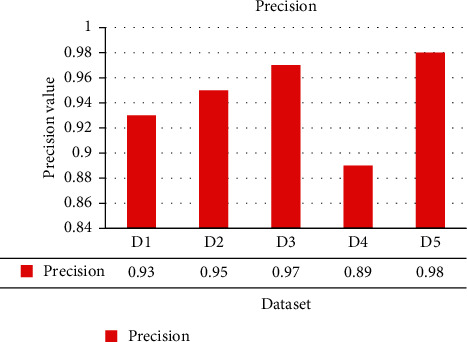
Precision comparison.

**Figure 4:**
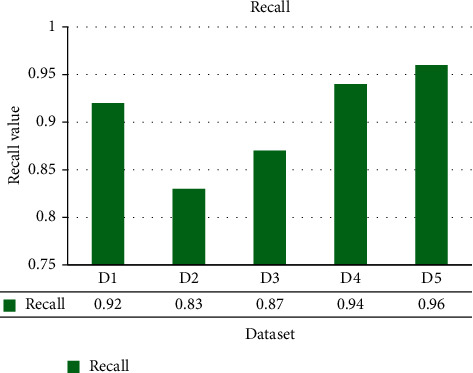
Recall comparison.

**Figure 5:**
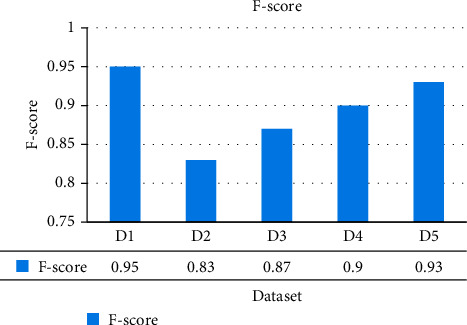
F-score comparison.

**Figure 6:**
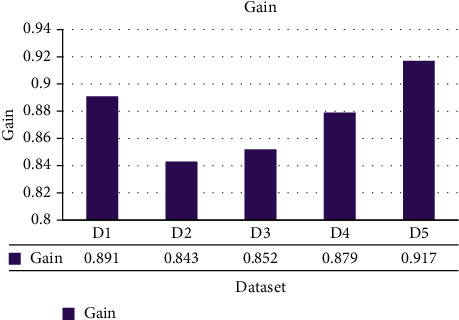
Gain comparison.

The ShoRAH method was tested for its capacity to determine the frequencies of particular clones in a population. Practically, every haplotypes were accurately reconstituted in the non-PCR-amplified data set, and their frequencies estimations were well associated with the grounded reality (Pearson's association coefficient *r*=0 : 92 for overall haplotypes and *r*=0 : 98 if outliers, in other words haplotypes with ≥1 mismatched, are eliminated). The PCR-amplified samples have a higher number of flawed haplotype matches and much more discrepant frequency estimations (*r*=0 : 89 for all and *r* =0 : 97 for every perfectly matched haplotypes), indicating that both haplotype reconstructing and frequency assessment are more complicated. For several haplogroups, accurate reconstructions and probability assessment were feasible, with frequencies as lower as 0.1 per cent for the PCR samples and 1 per cent for the non-PCR samples. The disparity in resolution could be described by the PCR and non-PCR samples having distinct mean coverage of 1050 and 3000 base pairs per sequencing location, correspondingly. The summation of every actual haplogroups we were capable to identify in every window was usually >99.5 per cent for the PCR data and >97.6 per cent for the non-PCR samples. Figures 3–6 show a comparative of the best precision, recall, F-score, and gain assessment parameters for each classification.

## 5. Discussion

Four of the six approaches tested used various Bloom filter variations to provide for filter compressing, storing of counted values, and representations of mapping in addition to collections [26]. Hash databases were employed in the other existing approaches, which did not produce false positives. Despite the fact that the storage effectiveness of the Bloom filter occurs at the expense of false positives, several significant error correction programmes have decreased or limited false-positive rates by adopting diverse techniques. The developers of these six applications worked hard to increase performance and reduce storage footprints while preserving or enhancing the accuracy of their corrections. Since efficiency and storage are no longer bottlenecking concerns that restrict the implementation of these technologies, we elected to focus entirely on rectification accuracy in this research.

Since correcting efficiency might be tested precisely, generated databases were employed. Only indirect evaluating measures (e.g., genome coverage of de novo assembly and N50 contig size, and percentage of mapping reads in genome alignments) may be obtained for effectiveness analysis when genuine experimental samples are employed. We suggest that using actual databases in instrument assessment could reveal insights that simulated research cannot deliver. However, considerable testing must be carried out on generated databases before going on to actual databases. The researchers of the six instruments studied in this research evaluated them utilizing both synthesized and actual databases. The effectiveness indicators for modelled and actual data sources are highly correlated. When contrasted to various well-known programmes, including as SGA, HiTEC, DecGPU, SHREC, Quake, Coral, and Reptile, Musket was continuously one of the best functioning correctors for all modelled and actual databases. We also showed that Musket outperformed Reptile in terms of efficiency measures. Whenever the developers of Lighter, Bless [27], and Trowel [28] compared their instruments, they reported that theirs outscored Musket by an insufficient margin. Musket, on the other hand, fared just and along with the other three techniques when it came to generated databases. Musket and Bloocoo are very equivalent, particularly in terms of the multistage error correction technique [29]. On a generated database with a 1 per cent error rate at 75 per cent coverage, they apparently obtained equal corrective efficiency as evaluated by recall and precision (refer to “Appendix Materials” in [29]). These two programmes performed similarly effectively on three databases in the existing investigation. Bloocoo, on the other hand, underperformed or collapsed on the three databases with higher reads (110 bp), implying that there are potentially bottleneck concerns in Bloocoo's scripting that restrict its implementation to extended reads.

The task of selecting ideal characteristics is an inherent problem in utilizing every corrector [30]. Only a limited programme feature automatic parametric selection that is responsive to the data sets being analysed. Bless, like the other instruments assessed in this work, may identify a suitable value for *M*, k-mer multiplicity thresholds, but it could not determine an ideal *k* (excluding for reptile, that selects *k* = *log*4*|G|*, wherein *G* is the genomic lengths). For every data set, we utilized KmerGenie [25] to find the best *k*. Although it is likely that the *k*  chosen by KmerGenie is not the best measure including all six applications assessed, we ran a few experiments altering *k* and other user-defined, tool-specific characteristics and found no significant differences in efficiency measures. For equivalent purposes, we established the read alignments edit distance at 3 (47/67-bp reads) or 5 (110-bp reads) depending on the 5 paper centre length suggestion. As a conclusion, we only presented the outcomes produced with the tool-specific characteristics adjusted to defaults, the selected *k* values, and the specified modification intervals.

## 6. Conclusion

Before proceeding with any further downstream in-depth assessment, it is critical to detect and repair flaws in NGS information. The goal of this comparison research was to offer an impartial and unbiased assessment of the impact of five NGS database properties on the effectiveness of k-spectrum-based error correction algorithms, with a focus on reliability. We discovered that genome size, coverage depth, and read length all influenced the effectiveness of the chosen approach.

## Data Availability

The data used to support the findings of this study are included in the article.
